# Biochemical Characterization of Three BLT Receptors in Zebrafish

**DOI:** 10.1371/journal.pone.0117888

**Published:** 2015-03-04

**Authors:** Toshiaki Okuno, Tohru Ishitani, Takehiko Yokomizo

**Affiliations:** 1 Department of Biochemistry, Juntendo University School of Medicine, Tokyo, Japan; 2 Department of Medical Biochemistry, Kyushu University, Fukuoka, Japan; 3 Division of Cell Regulation Systems, Department of Immunobiology and Neuroscience, Medical Institute of Bioregulation, Kyushu University, Fukuoka, Japan; Medical School of Hannover, GERMANY

## Abstract

The leukotriene B_4_ (LTB_4_) receptor 1 (BLT1) is a high affinity receptor for LTB_4_, a chemotactic and inflammatory eicosanoid. The LTB_4_ receptor 2 (BLT2) was originally identified as a low affinity receptor for LTB_4_, and, more recently, as a high affinity receptor for 12-hydroxyheptadecatrienoic acid (12-HHT). The zebrafish BLT receptors have not been previously identified and the *in vivo* functions of these receptors have been unknown. In this paper, we describe one zebrafish BLT1-like receptor, Blt1, and two zebrafish BLT2-like receptors, Blt2a and Blt2b. Cells expressing Blt1 exhibited LTB_4_-induced intracellular [Ca^2+^] increases, inhibition of cAMP production, ligand-dependent [^35^S]GTPγS binding, and transforming growth factor-α (TGFα) shedding activity in a dose-dependent manner, similar to human BLT1. Cells expressing Blt2a and Blt2b exhibited 12-HHT- and LTB_4_-induced intracellular [Ca^2+^] increases, inhibition of cAMP production, [^35^S]GTPγS binding, and TGFα shedding activity, with a dose-dependency similar to human BLT2. Reverse transcription (RT)-PCR analysis and whole-mount *in situ* hybridization revealed that *blt1, blt2a, blt2b*, zebrafish LTA_4_ hydrolase (*lta4h*), and zebrafish 5-lipoxiganase (*5lo*) are expressed in zebrafish embryos. Knockdown of *blt1* by morpholino antisense oligonucleotides resulted in delayed epiboly at gastrulation. Consistently, knockdown of *lta4h*, an enzyme mediating LTB_4_ production, induced a phenotype similar to knockdown of *blt1*. These results suggest that the LTB_4_-BLT1 axis is involved in epiboly in zebrafish development.

## Introduction

Leukotriene B_4_ (LTB_4_), an eicosanoid derivative of arachidonic acid metabolism produced by the sequential actions of 5-lipoxygenase (5-LO) and leukotriene A_4_ hydrolase (LTA_4_H), is a potent leukocyte chemoattractant [1]. Two G-protein-coupled receptors (GPCRs) for LTB_4_, BLT1 and BLT2, have been identified [2]. BLT1 is a high affinity LTB_4_ receptor [3], while BLT2 was originally identified as a low affinity LTB_4_ receptor [4]. Recently, we demonstrated that 12(*S*)-hydroxy-5-*cis*-8,10-*trans*-heptadecatrienoic acid (12-HHT), which had been considered as merely a by-product of thromboxane synthesis from prostaglandin endoperoxide, is an endogenous high affinity ligand for BLT2 [5,6]. BLT1 and BLT2 form a gene cluster on both human and mouse chromosomes, suggesting that these receptors were generated by a gene duplication machinery [4]. BLT1 is expressed on various immune cells including neutrophils, eosinophils [7], monocytes, dendritic cells [8,9], activated T-cells [10], and osteoclasts [11], and induces the activation and migration of these cells [12]. The *in vivo* role of BLT2 has not been established, but our recent work revealed that BLT2 has an anti-inflammatory function in a mouse model of inflammatory colitis [13], as well as a protective role in allergic airway inflammation [14]. BLT2 also promotes wound healing by accelerating keratinocyte migration [15].

The zebrafish has emerged as a useful model system for genetic and pharmacological analyses of embryogenesis because fertilization and embryo development occur outside the maternal body and the embryos are transparent [16,17]. In addition, the zebrafish has been used as a model for studying inflammation and immunity because the immune system is largely conserved between zebrafish and mammals. Bischel *et al*. have shown that LTB_4_ induces neutrophil migration into the fins of live zebrafish [18], and recent forward genetic screening in zebrafish larvae has revealed that the *lta4h* locus modulates susceptibility to mycobacterial infections [19,20]. However, BLT receptors have not been definitively identified in zebrafish. In this study, we identified genes for zebrafish BLT receptors by bioinformatic and biochemical analyses, and revealed an unexpected function of zebrafish BLT1 in embryogenesis.

## Material and Methods

### Materials

LTB_4_ and 12-HHT were purchased from Cayman Chemical (Ann Arbor, MI). Probenecid was purchased from Sigma-Aldrich (St. Louis, MO). Pluronic F-127, Alexa-488-conjugated anti-Rat IgG (Molecular Probes), and fetal calf serum (FCS; GIBCO) were obtained from Invitrogen (Carlsbad, CA). A penicillin-streptomycin solution and geneticin (G418) were purchased from Nacalai Tesque (Kyoto, Japan). [^35^S]-guanosine 5’-O-(gamma-thio) triphosphate (GTPγS) was obtained from Perkin-Elmer Life Science (Boston, MA). Anti-hemagglutinin (anti-HA; clone 3F10) was purchased from Roche (Penzberg, Germany).

### cDNA cloning and plasmid construction of zebrafish Blts

The cDNA of zebrafish *blt1*, *blt2a*, *blt2b* and *lta4h* were isolated by PCR from cDNA templates prepared from zebrafish embryos at 24–72 hours post fertilization (hpf). The following primers were used for cDNA cloning: *blt1* (5’- atggcaactcctttaactccggtc -3’ and 5’—tcaatgcagggggtcagagtcttg -3’), *blt2a* (5’- atggcgttgaaccttctgtccccc -3’ and 5’—tcactttccattattctggggtgcg -3’), *blt2b* (5’—atggcattggaaaatggcagcttctc -3’ and 5’—ttatagcctgatgacatccctagtc—3’), and *lta4h* (5’—atgactccagtttcagaccctagc—3’ and 5’—ctagccatcgattttcagatccttg—3’. The sequences for *blt1*, *blt2a* and *blt2b* were amplified by PCR and cloned into the pCXN2-HA vector [21]. The following primers were used: *blt1*, (5’- ttgcgatatcgcaactcctttaactccggtcttc -3’ and 5’—gcgaattctcaatgcaggggggtcagagtc -3’), *blt2a* (5’- ttgcgatatcgcgttgaaccttctgtccccctc- 3’ and 5’—gcgaattctcactttccattattctggggtgc-3’), and *blt2b* (5’—ttgcgatatcgcattggaaaatggcagcttctc-3’ and 5’—gcgaattcttatagcctgatgacatccctag- 3’). Zebrafish *blt1*, *blt2a*, and *blt2b* were amplified by PCR and cloned into the pCS2+ vector. Zebrafish *lta4h* and *5lo* were amplified by PCR and cloned into the pCS2P+ vector. The following primers were used: *lta4h* (5’—cggaattccaccatgactccagtttcagaccctagc—3’ and 5’—ccgctcgagctagccatcgattttcagatccttg—3’) and *5lo* (5’—cggaattcgcttgaaaatgcccagctacacgg—3’ and 5’—ccgctcgagttacacagccacactgtttgggattc—3’). All of the clones were verified by DNA sequencing.

### Cell culture, transfection, and cell sorting

Chinese hamster ovary (CHO) cells were maintained in Ham’s F-12 medium (Wako) containing 10% FCS, 100 units/ml penicillin, and 100 μg/ml streptomycin in 5% CO_2_ at 37°C. CHO cells were transfected with expression vectors using Lipofectamine LTX and the PLUS Reagent (Invitrogen) according to the manufacturer’s protocol. At 48 h post-transfection, the medium was changed to selection medium containing 1 mg/ml G418. After 2–3 weeks of selection, G418-resistant cells were stained with anti-HA (2 μg/ml) and Alexa-Fluor 488-conjugated anti-Rat IgG (10 μg/ml). Cells expressing the BLT receptors were collected as polyclonal populations by cell sorting using FACSAria II (Becton, Dickinson and Company, Franklin Lakes, NJ) and maintained in 0.3 mg/ml G418. A FACSCalibur instrument (Becton Dickinson) was used for flow cytometry.

### Calcium mobilization assay

CHO cells (3 × 10^4^) stably expressing one of the receptors were seeded onto 96-well plates. After 16 h, cells were loaded with 4 μM Fluo-8 AM (ABD Bioquest) in 100 μl of HP buffer (HBSS containing 2.5 mM probenecid and 20 mM HEPES, pH 7.4) supplemented with 0.04% Pluronic F-127 and 1% FCS at 37°C for 30 min, followed by a further incubation at room temperature for 30 min. The cells were washed twice with HP buffer and agonist-induced intracellular calcium mobilization was determined by monitoring the fluorescence intensity (excitation at 485 nm, emission at 525 nm) using a FlexStation 3 plate reader (Molecular Devices, Sunnyvale, CA).

### Membrane preparation and GTPγS binding assay

Cells were harvested and sonicated in ice-cold homogenization buffer (20 mM Tris-HCl, pH 7.4, 0.25 M sucrose, 10 mM MgCl_2_, and 2 mM EDTA) containing a protease inhibitor mixture (Nacalai Tesque, Kyoto Japan). The homogenate was centrifuged at 800 × g for 5 min at 4°C; the supernatant was collected and centrifuged at 100,000 × g for 1 h at 4°C. The resulting pellet was resuspended in the homogenization buffer. The membrane preparation (10 μg of protein) was incubated in 100 μl of GTPγS binding buffer (20 mM Tris-HCl, pH 7.5, 100 mM NaCl, 5 mM MgCl_2_, 1 mM EDTA, 1 mM DTT, 5 μM GDP and 0.1% BSA) containing 1 nM [^35^S]GTPγS with LTB_4_ or 12-HHT for 30 min at 30°C. To determine nonspecific binding, unlabeled GTPγS was added to the binding mixture to a final concentration of 10 μM. The bound [^35^S]GTPγS was separated from free [^35^S]GTPγS by rapid filtration through GF/C filters and washed with ~2 ml of ice-cold TMN buffer (10 mM Tris-HCl, pH 7.5, 25 mM MgCl_2_, and 100 mM NaCl). The radioactivity of the filters was determined using a Top Count scintillation counter (Packard Instrument Co.).

### cAMP assay

CHO cells (4 × 10^4^ cells/well) were plated on 96-well plates. On the following day, cells were washed twice with Krebs-Ringer Bicarbonate Buffer containing glucose (KRBG buffer: 0.49 mM MgCl_2_, 4.56 mM KCl, 120 mM NaCl, 0.7 mM Na_2_HPO_4_, 1.5 mM NaHPO_4_, 15 mM NaHCO_3_ and 10 mM glucose (pH 7.4)). The cells were incubated with stimulation buffer (KRBG buffer containing 0.75 mM 3-isobutyl-1-methylxanthine (IBMX)) for 10 min at room temperature and then with 20 μM forskolin and LTB_4_, or 12-HHT, for 15 min at 37°C. The incubation was terminated by the addition of lysis buffer (pH 7.3) supplied in the CatchPoint cAMP Fluoresecent Assay Kit (Molecular Devices Corporation), and the concentration of cAMP in the lysate was determined according to the manufacturer’s protocol.

### TGFα shedding assay

A TGFα shedding assay was performed as previously reported [22]. HEK cells (2 × 10^5^ cells/well) were seeded into 12-well plates and incubated for 24 h. The cells were then transfected with the GPCR expression vector, pCAGGS-Gα_q/i1_ and pSS-AP-TGFα. At 24 h post-transfection, the cells were detached and resuspended in Hank’s balanced salt solution (HBSS) containing 5 mM HEPES (pH 7.4) and then seeded in a 96-well plate. After a 30 min incubation for 37°C, cells were stimulated with ligand for 1 h. The conditioned medium was transferred into another 96-well plate and *p*-nitrophenyl phosphate (*p*-NPP) solution (10 mM p-NPP, 40 mM Tris-HCl (pH 9.5), 40 mM NaCl and 10 mM MgCl_2_) was added to both a conditioned medium plate and a cell plate. Absorbance at 405 nm (OD_405_) of both plates was read before and after a 1 h incubation at 37°C using a microplate reader (Bio-rad). To determine TGFα shedding, AP activity was calculated from the increase in OD405 in the conditioned medium (ΔOD_medium_) relative to the cell plate (ΔOD_cell_) as follows. AP activity in the conditioned medium (AP_media_) (%) was defined as the ratio of ΔOD_medium_ to total ΔOD values (ΔOD_medium_ plus ΔOD_cell_).

### Zebrafish maintenance, morpholino injection and RT-PCR

Zebrafish strain AB was maintained under standard conditions. All experimental animal care was performed in accordance with institutional and national guidelines and regulations. The study protocol was approved by the institutional review board of Kyushu University. (All the experiments were performed at Kyushu University). Morpholino antisense oligonucleotides (MO) were designed and obtained from Gene Tools (Philomath, OR, USA). The sequences of translation-blocking MO and splice-blocking MO against *blt1* were 5’—GGCCATTTGACTCAAACTTTATGGT- 3’ (*blt1* MO) and 5’—CTATTAGACATACCGATAAAATGGC—3’ (*blt1* spl MO), respectively. The translation-blocking MO against *lta4h* (*lta4h* MO) has been described previously [19]. To test the specificities of the *Blt1* MO and *lta4h* MO, the MO target sequences of *Blt1* and *lta4h* were amplified by PCR and cloned into the pCS2P-EGFP vector (kindly provided by A. Kawahara, University of Yamanashi). MOs (5 ng) were injected into zebrafish embryos at the one- or two-cell stage. Total RNA from control MO- or *blt1* spl MO-injected embryos was obtained at 24 hpf using the Trizol reagent (Invitrogen-Gibco, Carlsbad, CA) and was used as the template for generating cDNA (Superscript II reverse transcriptase; Invitrogen-Gibco). A 338 base pair (bp) *blt1* fragment was amplified using the following primers: *blt1*_fw, 5’—aggctgaggaccaagaagaggctcc—3’, and *blt1*_rv, 5’—ggcaaccagaagagggtgaaagcag—3’. The 392 bp nonsplicing transcript of *blt1* was amplified using the following primers: *blt1*_fw and *blt1*_rv2 5’—aatgtcggtgccgtcgcttattgat—3’. A 264 bp *blt2a* fragment was amplified using the following primers: *blt2a*_fw, 5’—tgggactttctgcacccgttttagc—3’, and *blt2a*_rv, 5’—gtccgaaggtgacgacaatcaccag—3’. A 299 bp *blt2b* fragment was amplified using the following primers: *blt2b*_fw, 5’—gcgtctgtactcgcagactgaccgt—3’, and *blt2b*_rv, 5’—ggcttgaatgaggttctcgcttcgt—3’. A 251 bp *lta4h* fragment was amplified using the following primers: *lta4h*_fw, 5’—gctccttctcctcgctctccaagtg—3’, and *lta4h*_rv, 5’—aaggcagggtgatttccaaagggg—3’. A 330 bp *5lo* fragment was amplified using the following primers: *5lo*_fw, 5’—tgaaaatgcccagctacacggtgac—3’, and *5lo*_rv, 5’—ctttgtcatcaaccaaccagcggaag—3’.

### Whole-mount *in situ* hybridization

Whole-mount *in situ* hybridization was performed according to a standard protocol. A digoxigenin-labeled antisense RNA probe was prepared using pCS2-*blt1*, pCS2-*blt2a*, pCS2-*blt2b*, and pCS2-*lta4h*. Capped mRNA was synthesized using an SP6 mMessage mMachine kit (Ambion, Austin, TX, USA) and purified using Micro Bio-Spin columns (Bio-Rad, Hercules, CA, USA).

### Statistical Analysis

Statistical analysis was performed using Prism (Graphpad Software) for all comparisons.

## Results

### Cloning of *blt1* and *blt2* homologs in zebrafish

**Fig 1:**
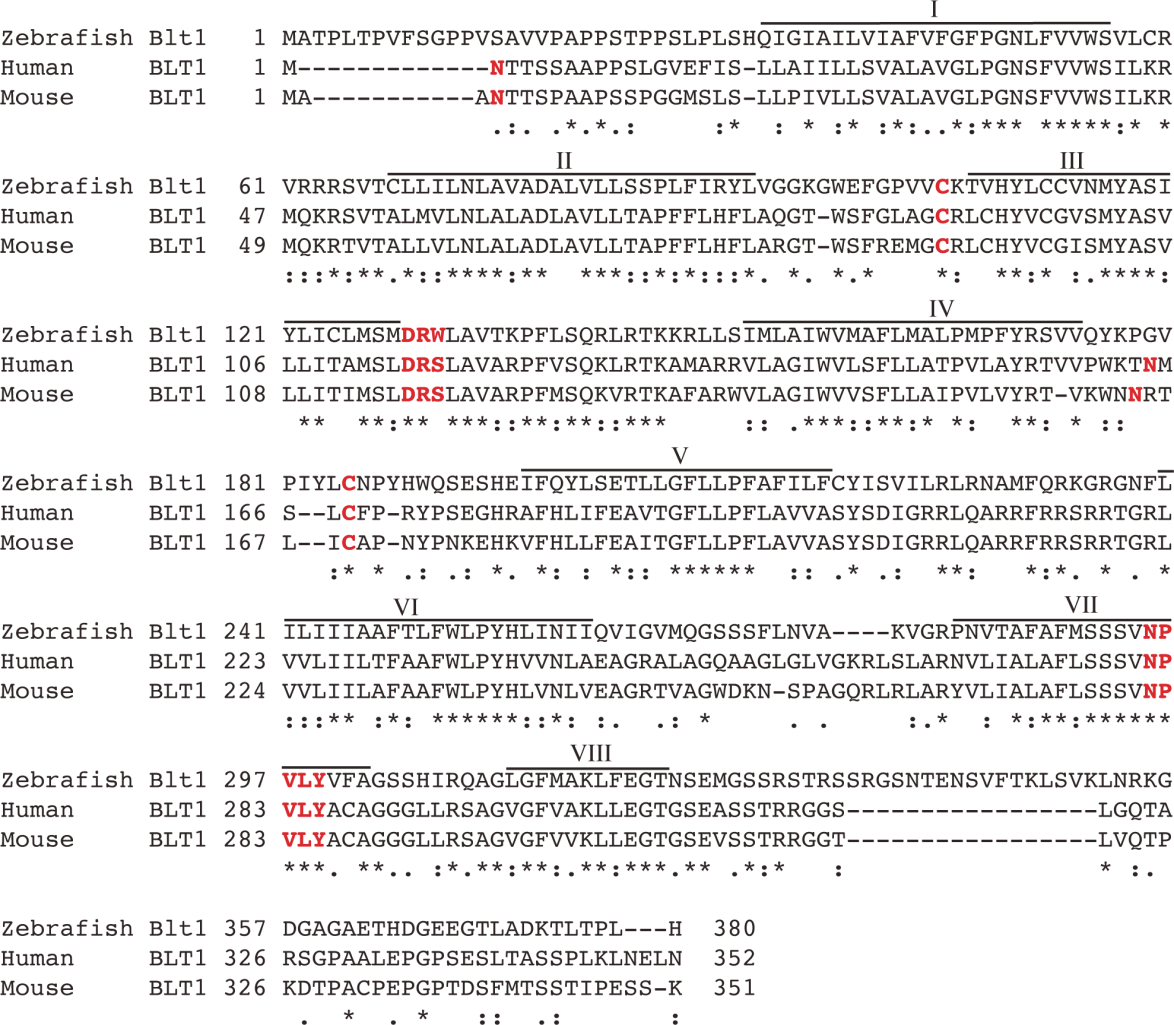
Sequence alignment of zebrafish Blt1 with human and mouse BLT1. The amino acid sequences of zebrafish Blt1 and human and mouse BLT1 were aligned using MAFFT (http://www.ebi.ac.uk/Tools/msa/mafft/). Identical residues are indicated with asterisks (*), highly conserved residues with colons (:), and semi-conserved residues with periods (.). The predicted glycosylated aspartate, the predicted cysteine residues that form disulfide bonds, and the DRY and N/DPxxY motifs are indicated in red characters. Putative helical domains that form helix I to helix VIII are indicated above the aligned sequences. The sequence of zebrafish Blt1 is available from EMBL/GenBank/DDBJ under accession no. LC009449.

**Fig 2:**
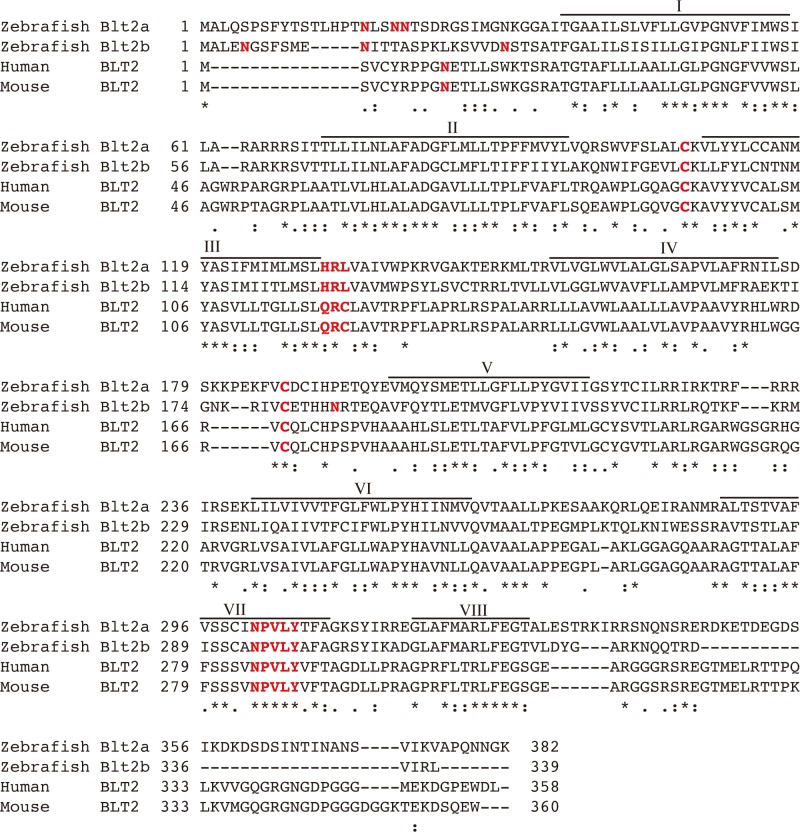
Sequence alignment of zebrafish Blt2a and Blt2b with human and mouse BLT2. The amino acid sequences of zebrafish Blt2 and human and mouse BLT2 were aligned using MAFFT (http://www.ebi.ac.uk/Tools/msa/mafft/). Identical residues are indicated with asterisks (*), highly conserved residues with colons, and semi-conserved residues with periods (.). The predicted glycosylated aspartate, the predicted cysteine residues that form disulfide bonds, and the DRY and N/DPxxY motifs are indicated in red. Putative helical domains that form helix I to helix VIII are indicated above the aligned sequences. The sequences of zebrafish Blt2 are available from EMBL/GenBank/DDBJ under accession nos. LC009450 and LC009451.

**Fig 3:**
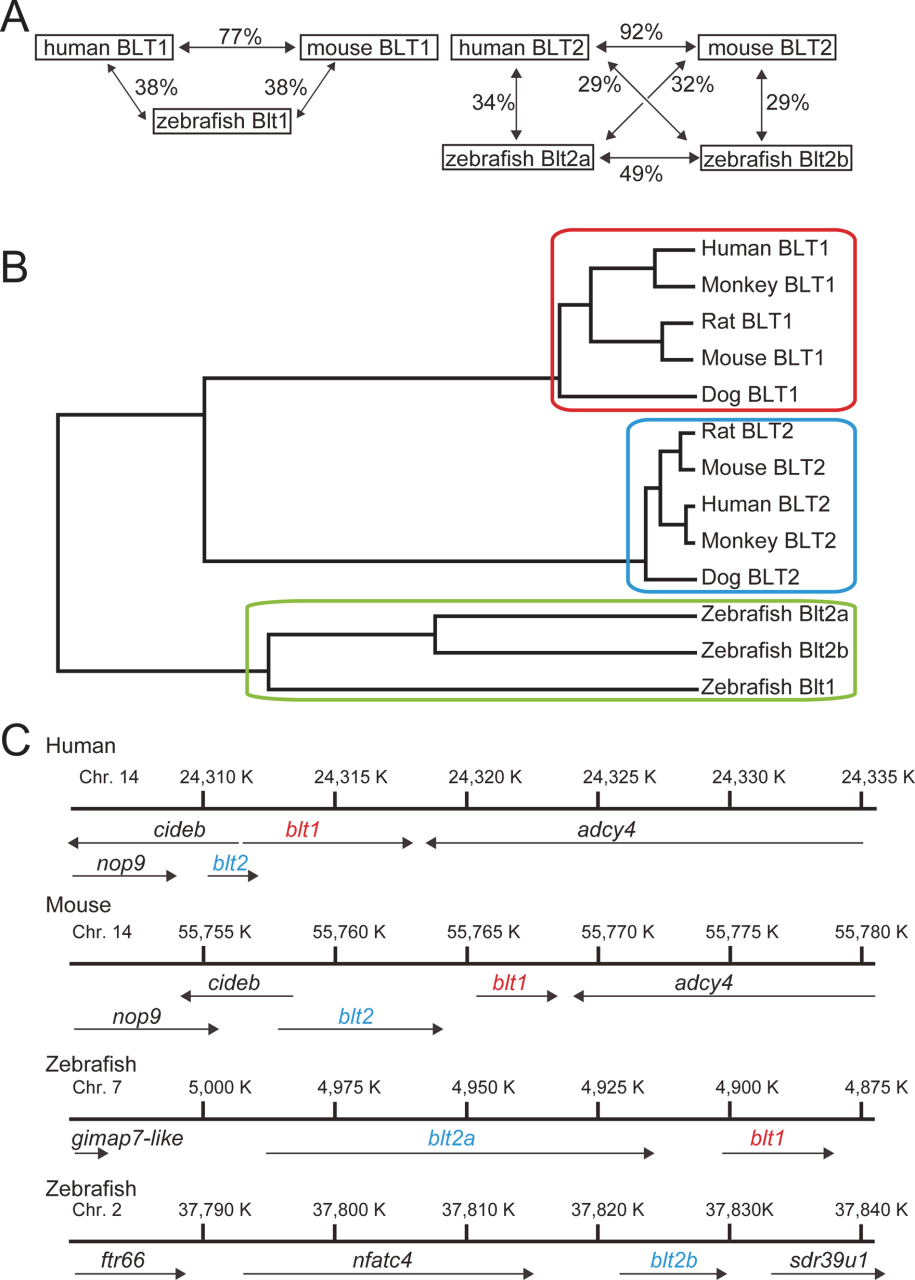
Sequence analysis of the zebrafish Blt receptors. (A) The amino acid identities among BLT1s (left) and BLT2s (right) are illustrated. (B) A phylogenic tree of BLT homologs in chordates was generated by GENETEX-MAC using the Unweighted Pair Group Method using the arithmetic Average (UPGMA). (C) Chromosomal locations of the human, mouse, and zebrafish *blt1* and *blt2* genes are shown. Chr., chromosome; *cideb*, cell death-inducing dffa-like effector b; *nop9*, nucleolar protein; *adcy4*, adenylate cyclase 4; *gimap7*-like, GTPase IMAP family member 7-like; *ftr66*, fin TRIM family, member 66; *nfatc4*, nuclear factor of activated T-cells, cytoplasmic, calcineurin-dependent 4; *sdr39u1*, short chain dehydrogenase/reductase family 39U.

To obtain cDNAs for BLT receptors in zebrafish, we searched for putative zebrafish BLT receptors and identified three BLT-like sequences (XP_002662767, XP_009301152, XP_003197923) from the NCBI database. We amplified full-length cDNAs corresponding to these three sequences and cloned each into an expression vector. From the results of the pharmacological experiments described below, XP_002662767, XP_009301152, and XP_003197923 were named *blt1*, *blt2a*, and *blt2b*, respectively. We aligned the amino acid sequences of zebrafish, human, and mouse BLT1 (Fig. 1), and found that they shared moderate homology, with sequence identities of 38% between zebrafish and human or mouse. We also aligned the amino acid sequences of zebrafish, human and mouse BLT2 (Fig. 2), and found that they shared relatively low homology with sequence identities of 29–34% between zebrafish and human or mouse (Fig. 3A). A phylogenic analysis showed that zebrafish BLTs form a clade independent from mammalian BLT1 and BLT2 (Fig. 3B). In human and mouse, the *blt1* and *blt2* genes are located on chromosome 14. In zebrafish, the *blt1* and *blt2a* genes are located on chromosome 7, and the *blt2b* gene is located on chromosome 2 (Fig. 3C).

### Ligand identification and intracellular signaling of Blt1, Blt2a, and Blt2b

**Fig 4:**
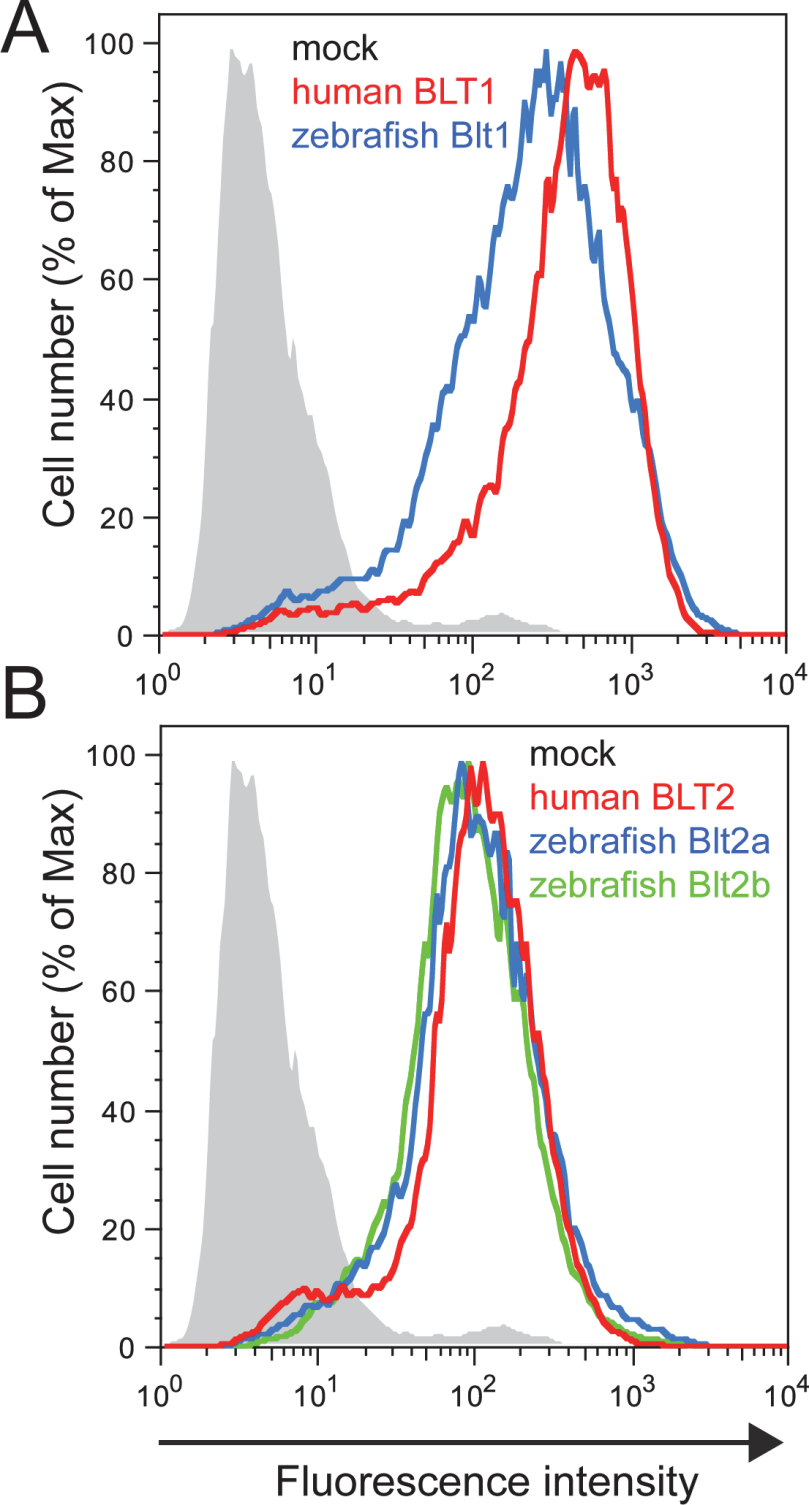
Establishment of CHO cells expressing the zebrafish Blt receptors. CHO cells stably expressing hBLT1, zebrafish Blt1, hBLT2, zebrafish Blt2a, or Blt2b were sorted after staining the cell surface Blts using an anti-HA antibody, and the surface expression was analyzed by flow cytometry.

**Fig 5:**
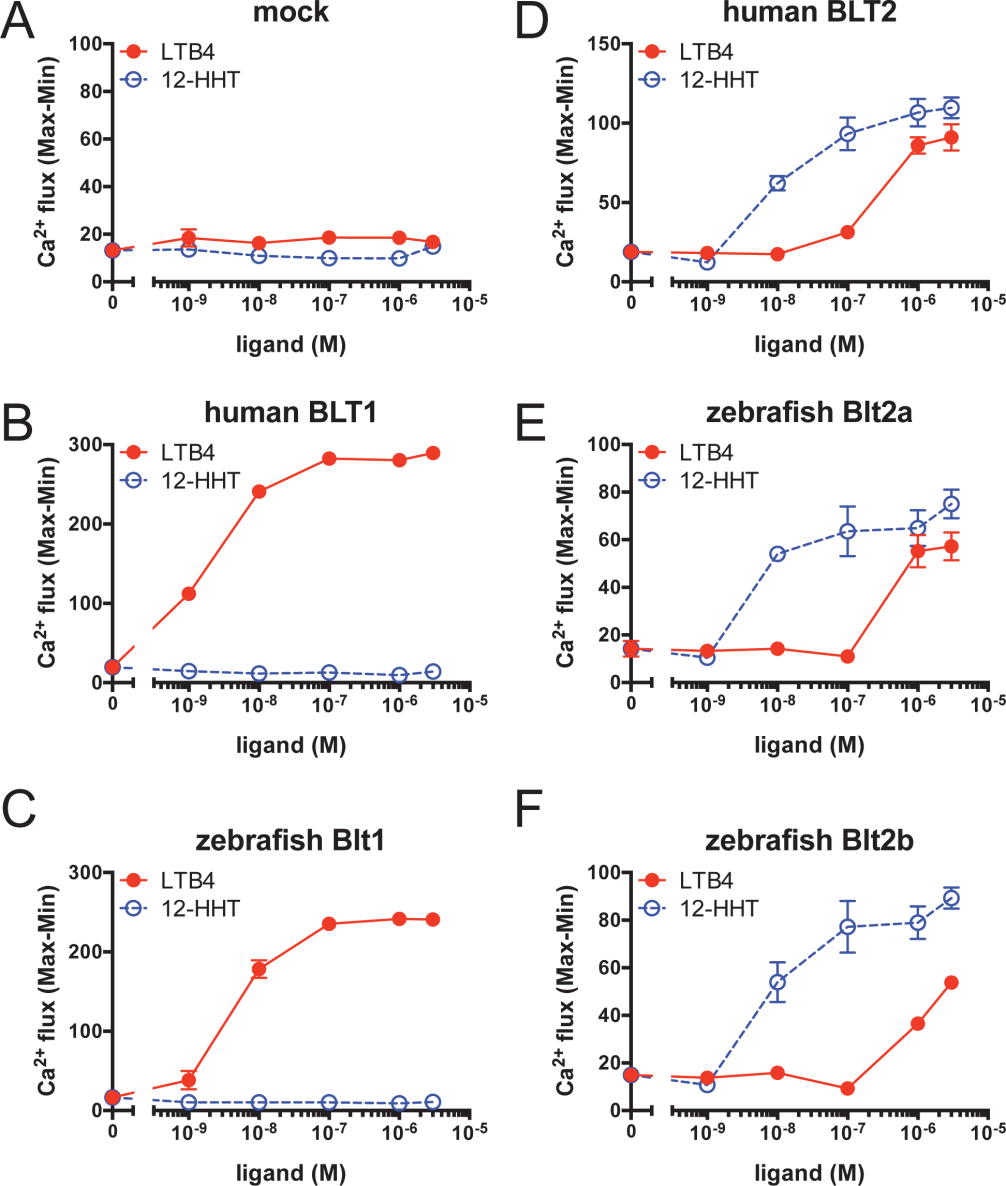
Calcium mobilization in CHO cells expressing the zebrafish Blt receptors. Intracellular calcium mobilization in CHO cells stably expressing hBLT1, zebrafish Blt1, hBLT2, zebrafish Blt2a, or Blt2b was analyzed using a FlexStation plate reader to measure fluorescence intensity. Data represent the mean ± s.e.m. (n = 4). These data are representative of at least two independent experiments with similar results.

To examine the ligands and intracellular signaling of zebrafish Blt1, Blt2a, and Blt2b, we established CHO cells stably expressing N-terminally HA-tagged zebrafish Blt1, Blt2a, and Blt2b, as well as human BLT1 (hBLT1) and BLT2 (hBLT2). CHO cells expressing the receptors were sorted as polyclonal populations and analyzed using a flow cytometer. The hBLT1 and zebrafish Blt1 were expressed on the cell surface at similar levels (Fig. 4A), and hBLT2, zebrafish Blt2a, and Blt2b were expressed on the cell surface at similar levels (Fig. 4B). Previously, we showed that human BLT1 and BLT2 are coupled to the Gi and Gq families of G-proteins [23–25]. To investigate intracellular signaling through zebrafish BLTs, we performed calcium mobilization assays using the transfected cells. In hBLT1 (Fig. 5B) and zebrafish Blt1 (Fig. 5C) cells, intracellular free calcium concentration increased in a dose-dependent manner in response to LTB_4_ stimulation, whereas 12-HHT did not induce calcium mobilization in these cells, suggesting that zebrafish Blt1 is a zebrafish ortholog of human BLT1. In hBLT2 (Fig. 5D), zebrafish Blt2a (Fig. 5E), and zebrafish Blt2b (Fig. 5F) cells, the intracellular free calcium exhibited a dose-dependent increase in response to either 12-HHT or LTB_4_ stimulation, and 12-HHT activated zebrafish Blt2a and Blt2b at lower doses than LTB_4_, suggesting that zebrafish Blt2a and Blt2b are zebrafish orthologs of hBLT2.

**Fig 6:**
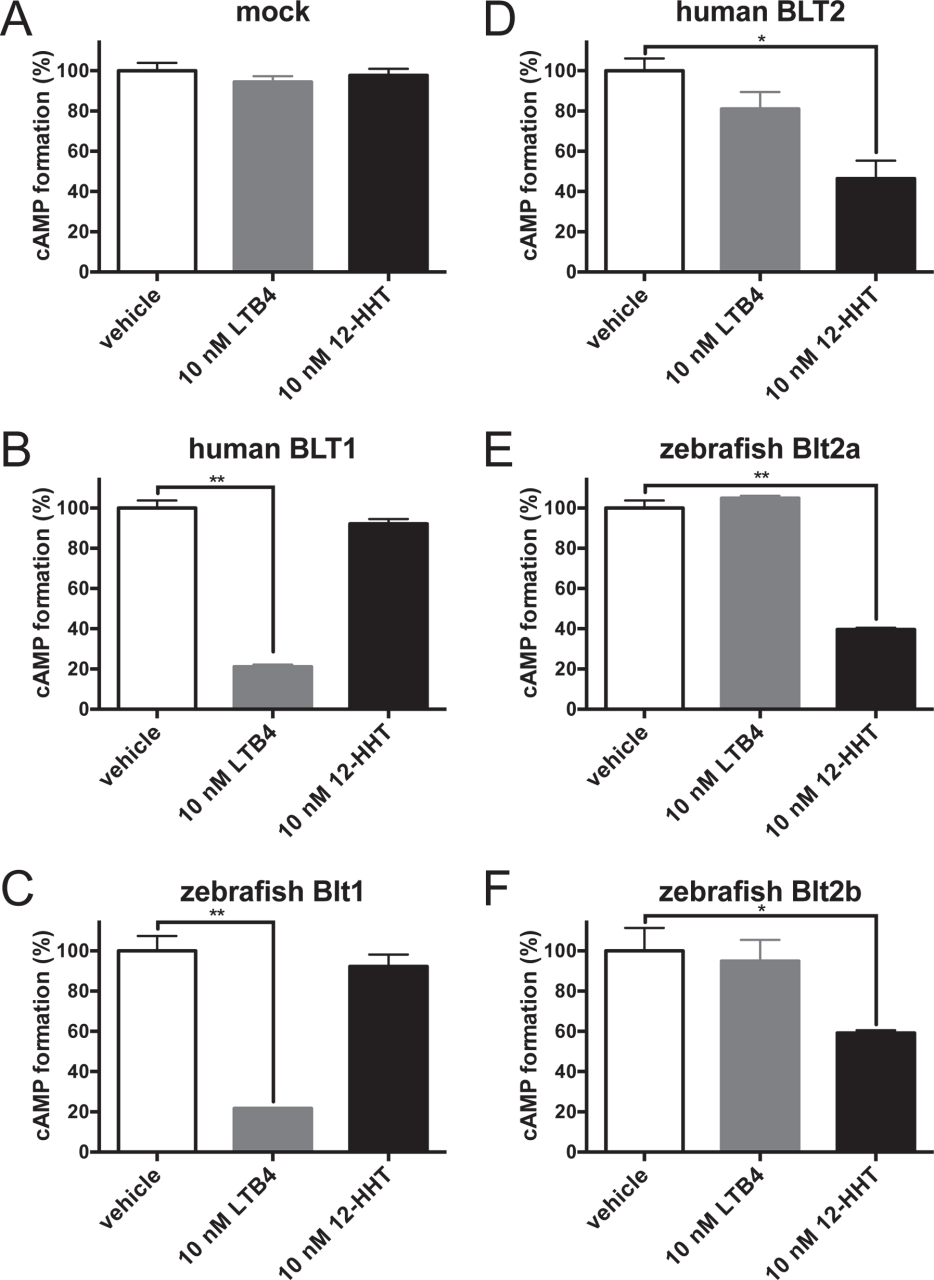
Levels of cAMP in CHO cells expressing the zebrafish Blt receptors. CHO cells stably expressing the human or zebrafish receptors were stimulated with 20 μM forskolin and 10 nM LTB_4_ or 12-HHT, and the levels of cAMP in cell lysates were determined. Data represent the mean ± s.e.m. (n = 3). **, P < 0.005; *, P < 0.05, one-way ANOVA with Bonferroni post-hoc test. These data are representative of at least two independent experiments with similar results.

**Fig 7:**
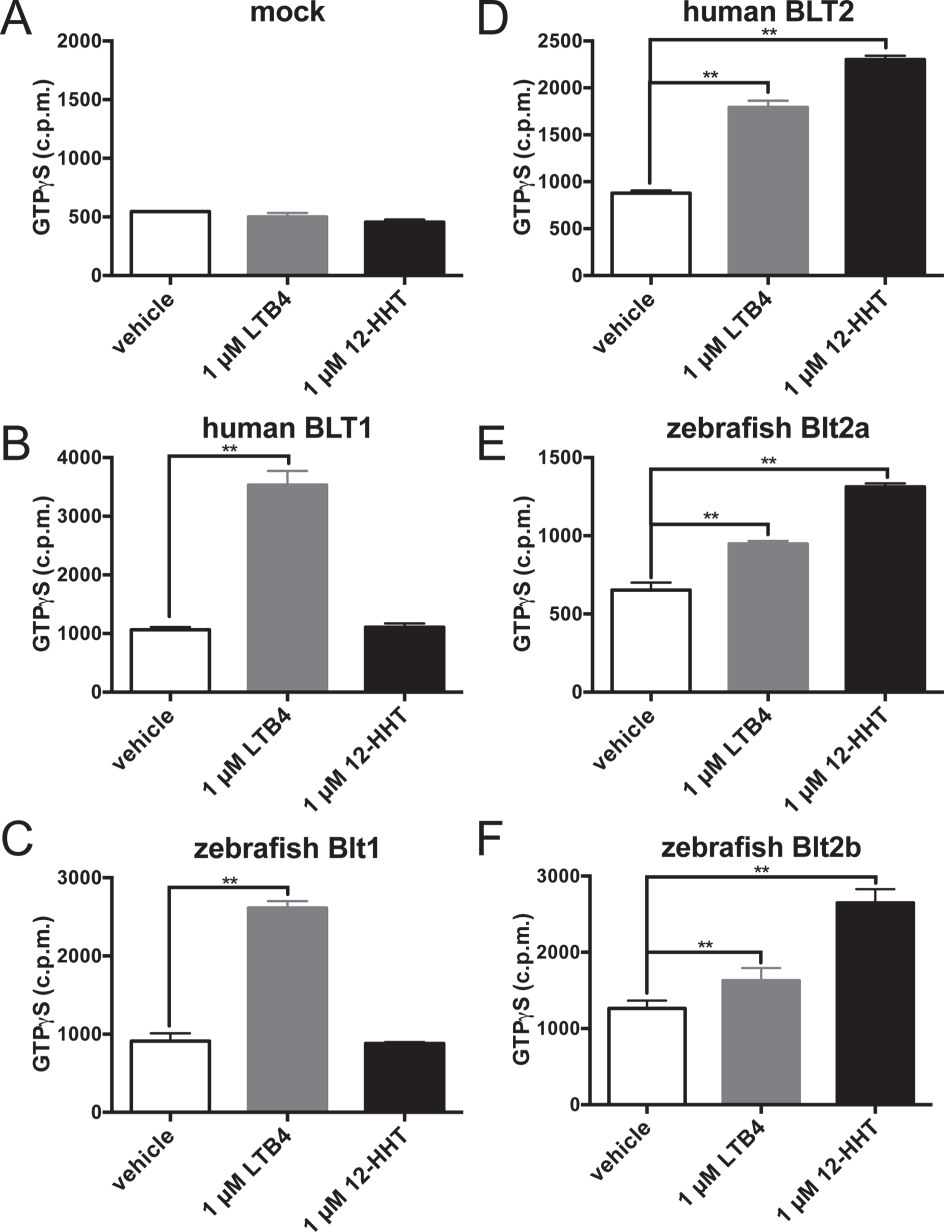
G-protein activity in CHO cells expressing the zebrafish Blt receptors. Membrane preparations of CHO cells expressing human or zebrafish receptors were incubated with 1 nM [^35^S] GTPγS and 1 μM LTB_4_ or 12-HHT, and specific binding was measured. Data represent the mean ± s.e.m. (n = 3). **, P < 0.005, one-way ANOVA with Bonferroni post-hoc test. These data are representative of at least two independent experiments with similar results.

Next, we quantified cAMP levels in CHO cells expressing the receptors. In hBLT1 (Fig. 6B) and zebrafish Blt1 (Fig. 6C) cells, 10 nM LTB_4_ inhibited forskolin-induced cAMP formation, but 12-HHT did not, suggesting that zebrafish Blt1 is a high affinity LTB_4_ receptor similar to hBLT1. In hBLT2 (Fig. 6D), zebrafish Blt2a (Fig. 6E), and zebrafish Blt2b (Fig. 6F) cells, 10 nM 12-HHT inhibited cAMP formation, but LTB_4_ had only a minimal effect on cAMP, suggesting that zebrafish Blt2a and Blt2b are high affinity 12-HHT receptors similar to hBLT2. To confirm that zebrafish BLT receptors directly activated G-proteins, we performed GTPγS binding assays using a membrane preparation of CHO cells expressing the receptors. Incubation with 1 μM LTB_4_ induced robust GTPγS binding, but 12-HHT did not, in hBLT1 (Fig. 7B) and zebrafish Blt1 (Fig. 7C) cells. Incubation with 1 μM 12-HHT induced robust GTPγS binding, and 1 μM LTB_4_ induced moderate but significant GTPγS binding, in hBLT2 (Fig. 7D), zebrafish Blt2a (Fig. 7E), and zebrafish Blt2b (Fig. 7F) cells. These results suggest that zebrafish Blt1 is a BLT1-type receptor, and zebrafish Blt2a and Blt2b are BLT2-type receptors.

### TGFα shedding activities of zebrafish Blt1, Blt2a, and Blt2b

**Fig 8:**
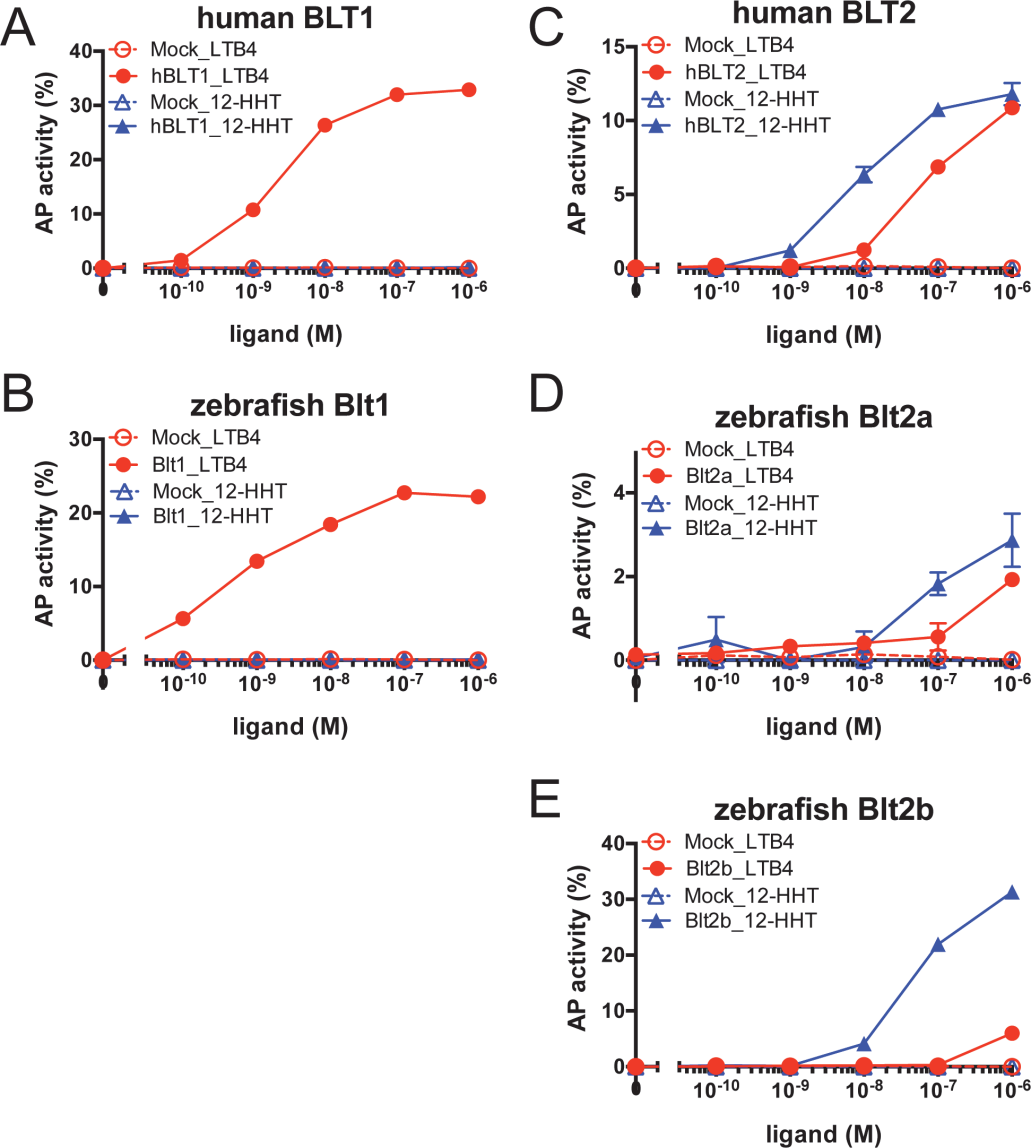
Ligand-dependent TGFα release via zebrafish Blt receptors. HEK293 cells expressing BLT receptors, Gα_q/i1_, and AP-TGFα were stimulated with LTB_4_ or 12-HHT. AP-TGFα release (%) was quantified using a colorimetric AP assay using *p*-NPP as a substrate. Data are representative of at least two independent experiments with similar results.

To confirm the ligands and intracellular signaling of zebrafish BLT receptors, we performed a TGFα shedding assay. In the TGFα shedding assay, GPCR activation is measured by the ectodomain shedding of a membrane-bound pre-form of alkaline phosphatase-tagged TGFα (AP-TGFα) into the medium [22]. HEK293 cells were transfected with a GPCR expression vector, a G_q/i1_ chimeric plasmid, and an expression plasmid encoding AP-TGFα, and then stimulated with a ligand, which resulted in the accumulation of released AP-TGFα into the medium (conditioned medium). LTB_4_ stimulation resulted in a dose-dependent increase in TGFα shedding that was similar between hBLT1 (Fig. 8A) and zebrafish Blt1 (Fig. 8B) cells. In hBLT2 (Fig. 8C), zebrafish Blt2a (Fig. 8D), and Blt2b (Fig. 8E) cells, TGFα shedding activity was dose-dependently increased by either 12-HHT or LTB_4_ stimulation. The lower AP activity of zebrafish Blt2a (Fig. 8D) may be affected by the lower expression of Blt2a on the cell surface in this assay (data not shown). In zebrafish Blt2b cells (Fig. 8E), the TGFα shedding activity induced by LTB_4_ was much lower than that induced by 12-HHT. Thus, Blt2b may exhibit greater specificity for 12-HHT than that shown by hBLT2.

### Expression of *blts*, *lta4h*, and *5lo* in zebrafish embryos

**Fig 9:**
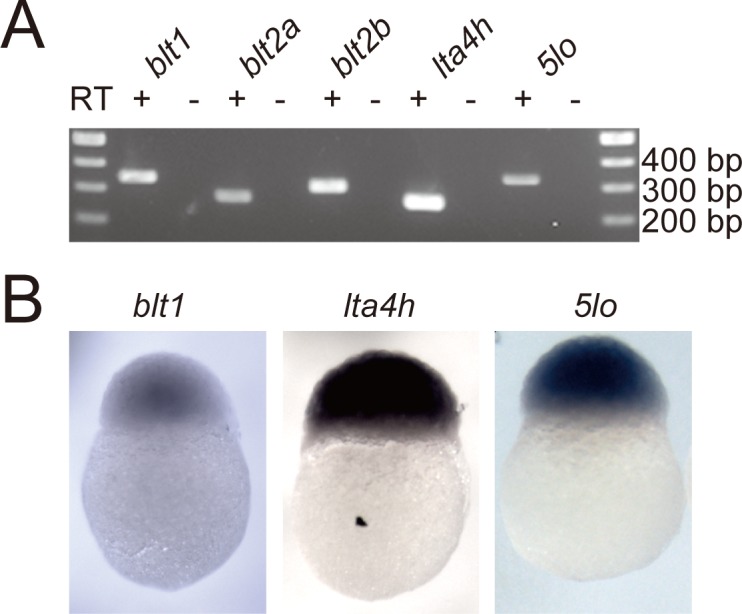
Expression of *blt1*, *blt2a*, *blt2b*, *lta4h*, and *5lo* in zebrafish embryos. (A) Expression of *blt1*, *blt2a*, *blt2b*, *lta4h*, and *5lo* mRNA were analyzed by RT-PCR. Total RNA was obtained from 24 hpf embryos and analyzed using specific primers for *blt1*, *blt2a*, *blt2b*, *lta4h*, and *z5lo*. (B) Expression of *blt1*, *lta4h*, and *5lo* mRNA was analyzed by whole-mount *in situ* hybridization. Zebrafish embryos (3 hpf) were hybridized with antisense probes.

To confirm the gene expression of *blt1*, *blt2a*, *blt2b*, *lta4h*, and *5lo* in zebrafish embryos, reverse transcriptase (RT)-PCR was performed using mRNA of zebrafish embryos at 24 hpf. The mRNAs of *blt1*, *blt2a*, *blt2b*, *lta4h*, and *5lo* were detected in zebrafish embryos (Fig. 9A) and whole-mount *in situ* hybridization revealed that *blt1*, *lta4h*, and *5lo* were widely expressed at 3 hpf (Fig. 9B).

### The LTB_4_-BLT1 axis is required for epiboly

**Fig 10:**
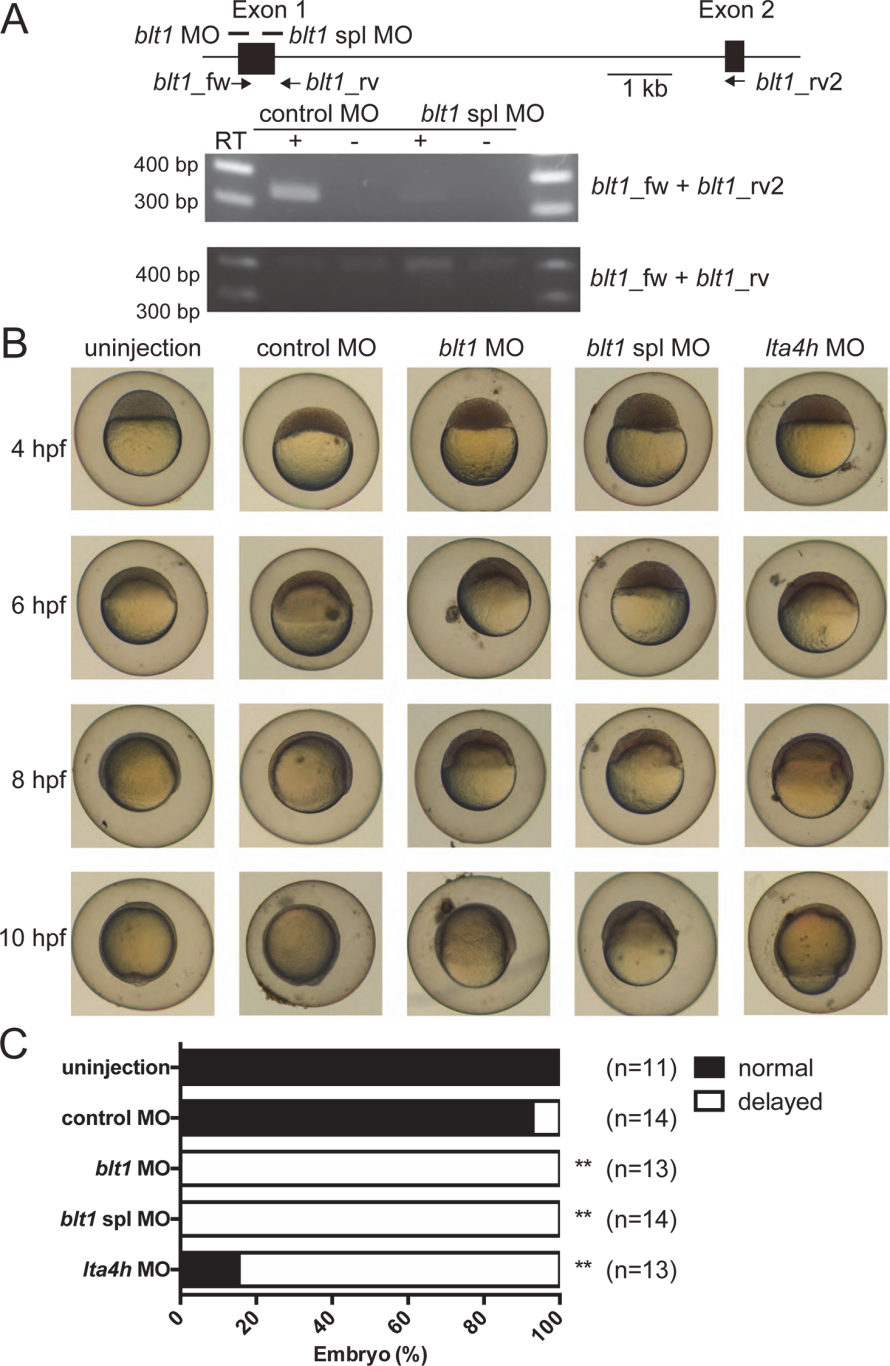
Morpholino-mediated knockdown of *blt1* and *lta4h* affects epiboly. (A) Diagram of a partial map of *blt1* genomic DNA. Exons and introns are shown as boxes and lines, respectively. Two nonoverlapping *blt1* MOs, *blt1* MO and *blt1* spl MO, were designed to target translation and splicing of Blt1, respectively. The *blt1* MO should eliminate the transcription of exon 1, which contains the translation start site, resulting in aberrant protein synthesis. The efficacy of *blt1* spl MO was validated by RT-PCR using *blt1*_fw and *blt1*_rv or *blt1*_rv2. Total RNA was isolated from *blt1* spl MO or control MO-injected embryos at 24 hpf. Reduced expression of normal spliced *blt1* transcripts and increased mis-spliced *blt1* transcripts are shown. (B) Representative images of delayed epiboly of *blt1* and *lta4h* morphants from 4 to 10 hpf. Embryos were injected with 2.5 ng MOs at the 1- to 2-cell stage. (C) Histogram illustrating the percentages of normal and delayed embryos. **, P < 0.005, one-way ANOVA with Bonferroni post-hoc test. Data are representative of at least two independent experiments with similar results.

To investigate the roles of Blt1 in zebrafish embryogenesis, we used two *blt1* morpholino antisense oligonucleotides: *blt1* MO blocks the translation of mature mRNAs and *blt1* spl MO blocks the normal splicing of *blt1* (Fig. 10A). We confirmed the efficiency and specificity of the *blt1* MO using a modified EGFP construct harboring the morpholino target sequence upstream of the respective start codon (S1 Fig. A and B). To confirm the knockdown of *blt1* by the *blt1* spl MO, we performed RT-PCR analysis using primer sets that amplify normal transcripts (*blt1*_fw and *blt1*_rv2, Fig. 10A) and abnormal transcripts with un-spliced introns (*blt1*_fw and *blt1*_rv, Fig. 10A). Injection of the *blt1* spl MO dramatically reduced the correctly spliced *blt1* transcripts (Fig. 10A, middle) and increased levels of the abnormal transcripts (Fig. 10A, low). These results suggested that the *blt1* spl MO efficiently knocked down *blt1* in zebrafish. We confirmed the efficiency and specificity of an *lta4h* MO using a modified EGFP construct harboring the morpholino target sequence upstream of the respective start codon (S1 Fig. C and D).

Interestingly, injection of either the *blt1* MO, *blt1* spl MO, or *lta4h* MO, but not injection of a control MO, resulted in a severe delay in epiboly during gastrulation (Fig. 10B and 10C). These results suggest that the LTB_4_-BLT1 axis is required for normal epiboly in zebrafish development.

## Discussion

In this study, we identified and characterized one zebrafish BLT1-like receptor and two zebrafish BLT2-like receptors. Knockdown of *blt1* delayed epiboly at gastrulation of zebrafish embryos. Database screening by sequence homology to human BLT1 and BLT2 identified three putative zebrafish Blts (Fig. 1–3) that share relatively low homologies to human and mouse BLT1 (~40%, Fig. 3A and 3B). Crystal structures of BLT1 and BLT2 have not been reported, but a structural model of the ligand binding site of BLT1 has been [26]. Alanine substitution of residues predicted to be potential ligand contact points in human BLT1, H94, Y102 (helix III), R156 (helix IV), E185 (helix V), and N241 (helix VI) resulted in reduced binding affinity, and all of these residues are conserved among zebrafish Blt1 and human and mouse BLT1 (Fig. 1). The R156A mutant of human BLT1 failed to show any [^3^H]LTB_4_ binding; R156 is the predicted binding site for the carboxyl group of LTB_4_ [26]. Among zebrafish Blt2a and Blt2b and human and mouse BLT2, all of these amino acids with the exception of H94 are conserved (Fig. 2), suggesting that H94 in helix III may be important in distinguishing between the chemical structures of LTB_4_ and 12-HHT. An evolutionary tree (Fig. 3B) suggests that the gene duplication of BLT1 and BLT2 occurred after the fish branched off from the other vertebrates. The presence of two BLT2-type receptors in zebrafish suggests that BLT2 may have specific roles in zebrafish that are not required in mammals.

To identify the ligands of putative zebrafish BLTs, we constructed expression vectors for the zebrafish Blts and conducted experiments to monitor GPCR-dependent signaling (Fig. 5–8). Previously, we found that the level of BLT1 on the plasma membrane is always higher than BLT2 in various cultured cells when overexpressed under the same promoter. Both human BLT1 and zebrafish Blt1 were expressed on the cell surface at a higher level than human BLT2, or zebrafish Blt2a or Blt2b (Fig. 4). The GPCR assays conducted here all suggest that Blt1 is a zebrafish ortholog of BLT1, and that Blt2a and Blt2b are zebrafish orthologs of BLT2 (Fig. 5–8). RT-PCR and whole-mount *in situ* hybridization of zebrafish embryos showed that mRNAs of *blt1*, as well as *lta4h* and *5lo*, enzymes mediating LTB_4_ production, were detected in zebrafish embryos (Fig. 9). The *blt1* and *lta4h* knockdown experiments indicated that the LTB_4_-Blt1 axis is involved in epiboly at gastrulation (Fig. 10).

Gastrulation involves a series of coordinated cell movements that establish the germ layers and the major body axes of the embryos [27]. Epiboly, which involves the thinning and spreading of a multilayered cell sheet, is the first coordinated cell movement of zebrafish gastrulation and occurs after the ninth or tenth zygotic cell division. Epiboly is visualized as the thinning and spreading of the blastoderm over the yolk. Since epiboly is initiated prior to the other cell movements in zebrafish gastrulation, the initial events in gastrulation can be studied in isolation from later, more complex cell movements [28–30]. Numerous molecules are involved in epiboly including microtubules, microfilaments, cell adhesion proteins, kinases, and transcription factors [27]. The prostaglandin biosynthetic enzymes cyclooxygenase-1 (Cox-1) and prostaglandin E_2_ synthase (Ptges) are also involved in epiboly. Treatment with 50 μM of indomethacin caused gastrulation arrest at 50% epiboly stage and 25 μM of indomethacin resulted in milder defect. Indomethacin-dependent epiboly defects were rescued by co-incubation with 1 μM PGE_2_ [31,32].

Lin *et al*. reported that Gα_12/13_ regulates epiboly formation through two distinct mechanisms: limiting E-cadherin activity and modulating the organization of the actin cytoskeleton [33,34]. In mammals, BLT1 activates small GTPases and induces the reorganization of actin cytoskeleton [35–37]; BLT1 activates leukocyte migration and firm adhesion to endothelial cells [10,38]. Thus, BLT1 may modulate the organization of the actin cytoskeleton and mediate cell migration in epiboly of zebrafish embryo.

We were not able to rescue the delay in epiboly by co-injection of MOs and *blt1* mRNA, suggesting that the localization and levels of Blt1 expression may be critical for proper epiboly. Although further analyses are required to understand the *in vivo* roles of Blt1 in epiboly, the molecular identification of zebrafish Blts will be useful for studying the *in vivo* roles of these receptors in zebrafish.

## Supporting Information

S1 Fig Specificity of *blt1* and *lta4h* morpholinos. (A) 5’ sequence of EGFP mRNA designed to evaluate the function of the *blt1* MO. The underline indicates the *blt1* MO target sequence, the start codon is highlighted in blue, and the beginning of the open reading frame (ORF) of EGFP is highlighted in green. (B) Representative images of the effects of the *blt1* MO on the expression of control EGFP and *blt1* MO-EGFP. Translation of EGFP mRNA containing the *blt1* MO target sequence is blocked by the *blt1* MO. Control EGFP is not blocked by the *blt1* MO. Embryos were injected with a mix of mRNA (250 pg) and MO (2.5 ng) at the one cell stage. EGFP fluorescence and bright-field (BF) images were taken at 7 hpf. (C) 5’ sequence of EGFP mRNA designed to evaluate the function of the *lta4h* MO. The underline indicates the *lta4h* MO target sequence, the start codon is highlighted in blue, and the beginning of the ORF of EGFP is highlighted in green. (D) Representative images of the effects of the *lta4h* MO on the expression of the *lta4h* MO-EGFP. Translation of EGFP mRNA containing the *lta4h* MO target sequence is blocked by the *lta4h* MO but not by a control MO. Embryos were injected with a mix of mRNA (250 pg) and MO (2.5 ng) at the one cell stage. EGFP fluorescence and BF images were taken at 6.5 hpf.
(TIF)
