# Supplementary figures and images for: Biochemical Characterization of Three BLT Receptors in Zebrafish
Source: PLoS One. 2015 Mar 4;10(3):e0117888. doi: 10.1371/journal.pone.0117888 (PMC4349892; doi:10.1371/journal.pone.0117888)

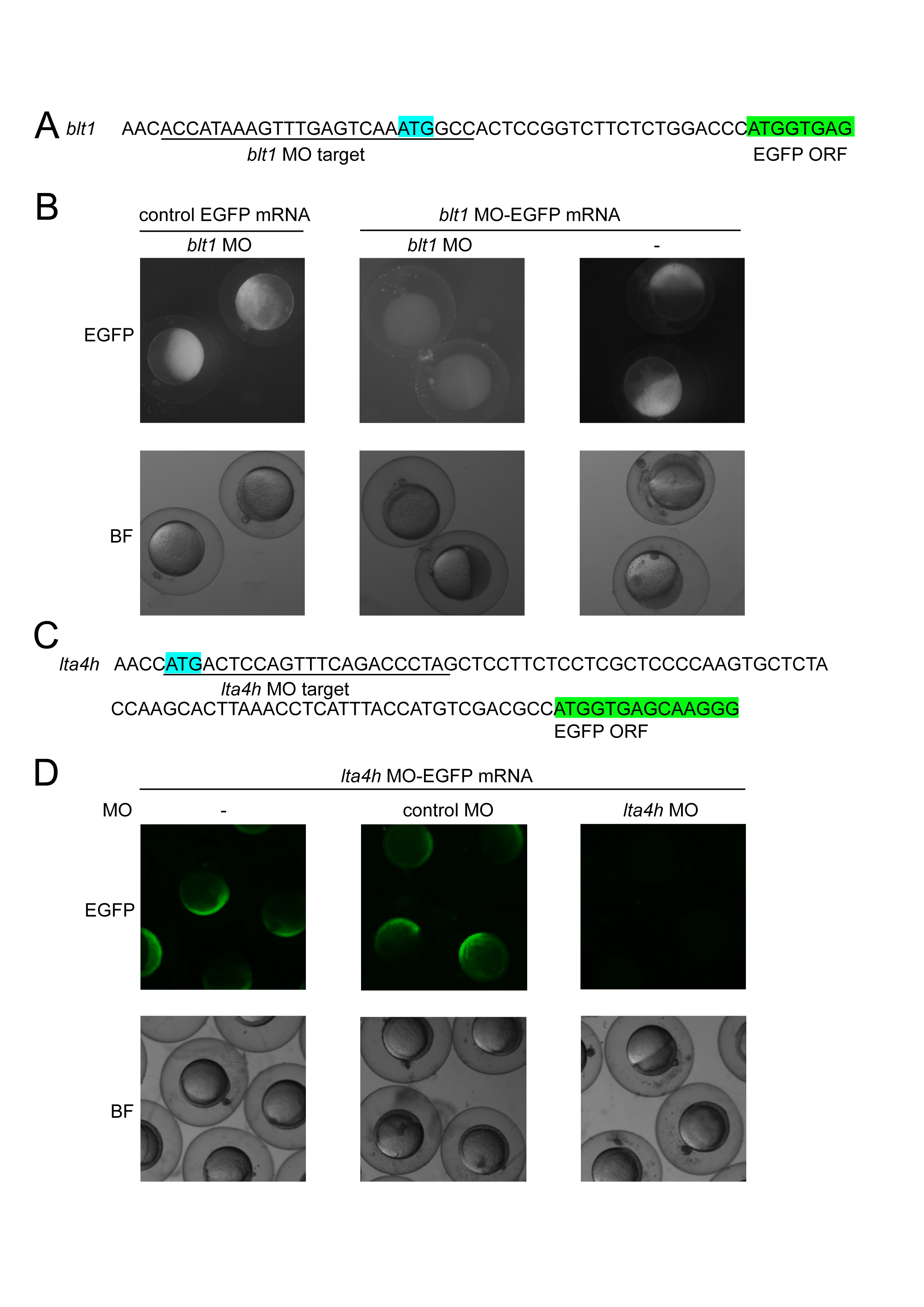

Supplement: S1 Fig — (A) 5’ sequence of EGFP mRNA designed to evaluate the function of the blt1 MO. The underline indicates the blt1 MO target sequence, the start codon is highlighted in blue, and the beginning of the open reading frame (ORF) of EGFP is highlighted in green. (B) Representative images of the effects of the blt1 MO on the expression of control EGFP and blt1 MO-EGFP. Translation of EGFP mRNA containing the blt1 MO target sequence is blocked by the blt1 MO. Control EGFP is not blocked by the blt1 MO. Embryos were injected with a mix of mRNA (250 pg) and MO (2.5 ng) at the one cell stage. EGFP fluorescence and bright-field (BF) images were taken at 7 hpf. (C) 5’ sequence of EGFP mRNA designed to evaluate the function of the lta4h MO. The underline indicates the lta4h MO target sequence, the start codon is highlighted in blue, and the beginning of the ORF of EGFP is highlighted in green. (D) Representative images of the effects of the lta4h MO on the expression of the lta4h MO-EGFP. Translation of EGFP mRNA containing the lta4h MO target sequence is blocked by the lta4h MO but not by a control MO. Embryos were injected with a mix of mRNA (250 pg) and MO (2.5 ng) at the one cell stage. EGFP fluorescence and BF images were taken at 6.5 hpf. (TIF) [file pone.0117888.s001.tif]
